# The Impact of Whole Grain Intake on Gastrointestinal Tumors: A Focus on Colorectal, Gastric, and Esophageal Cancers

**DOI:** 10.3390/nu13010081

**Published:** 2020-12-29

**Authors:** Valentina Tullio, Valeria Gasperi, Maria Valeria Catani, Isabella Savini

**Affiliations:** Department of Experimental Medicine, Tor Vergata University of Rome, 00133 Rome, Italy; valentinatullio.nu@gmail.com (V.T.); catani@uniroma2.it (M.V.C.); savini@uniroma2.it (I.S.)

**Keywords:** dietary fiber, esophagus, stomach and colorectal cancer, nutrition, polyphenols, refined grains, whole grains

## Abstract

Cereals are one of staple foods in human diet, mainly consumed as refined grains. Nonetheless, epidemiological data indicate that whole grain (WG) intake is inversely related to risk of type 2 diabetes, cardiovascular disease, and several cancer types, as well as to all-cause mortality. Particularly responsive to WG positive action is the gastrointestinal tract, daily exposed to bioactive food components. Herein, we shall provide an up-to-date overview on relationship between WG intake and prevention of gastrointestinal tumors, with a particular focus on colorectal, stomach, and esophagus cancers. Unlike refined counterparts, WG consumption is inversely associated with risk of these gastrointestinal cancers, most consistently with the risk of colorectal tumor. Some WG effects may be mediated by beneficial constituents (such as fiber and polyphenols) that are reduced/lost during milling process. Beside health-promoting action, WGs are still under-consumed in most countries; therefore, World Health Organization and other public/private stakeholders should cooperate to implement WG consumption in the whole population, in order to reach nutritionally effective intakes.

## 1. Introduction

Cereals, plant species belonging to the *Poaceae* family, are grasses producing edible grains (wheat, corn, rice, oats, barley, rye, millet, teff, sorghum, canary seed, triticale, and Job’s tears). Usually, the term also includes grains from non-herbaceous plants, known as pseudocereals (quinoa, buckwheat, amaranth, and wild rice), which have a composition similar to “real” grains [1,2,3].

Rice, maize, and wheat are the most common farmed cereals with a production of 2646 million tons in 2018–2019 [4]. As a primary source of carbohydrates, cereals provide about 60% of food energy worldwide and are mostly consumed as refined grains (RGs). However, health effects of cereals are mainly attributed to whole grains (WGs), and therefore governmental authorities are increasingly encouraging WG consumption.

Scientific interest in health properties of WGs began in the late 1970s, when the surgeon Denis Parsons Burkitt, noting the difference in disease incidence in rural Africa and the UK, brought together data coming from several disciplines and launched the dietary fiber hypothesis; he and other researchers, indeed, noted that a diet highly refined and lacking WG foods might be involved in several diseases, including coronary heart disease, obesity, diabetes, dental caries, as well as in some cancer types, such as gastric and colon tumors [5]. Since then, both epidemiological and interventional studies have reported potential health effects of unrefined grains [6,7,8,9,10,11,12,13,14]. Nonetheless, most of the population does not consume WGs, much likely due to several factors, including lack of nutritional education programs, low variety and palatability of WG-containing products, poor identification, and high purchase costs of WG foods. In addition, national dietary guidelines generally provide qualitative statements, such as “choose WG versions/varieties” or “increase WG intake”, and only few countries provide quantitative recommendations: i.e., 48 g/day (corresponding to 3 servings/day) in USA [15], ≥75 g/day in Denmark [16], and 70–90 g/day in Norway and Sweden [17,18]. In addition, among WG consumers, daily intake of unrefined grains is still below recommended levels, except for few countries, such as Denmark [19]. As emerged from National Health and Nutrition Examination Survey (NHANES, 2001–2012), mean WG intakes are 15.52 g/day for adults and 11.84 g/day for children in USA, and less than 8.0 and 1.0% of adults and children, respectively, meets WG recommendations [20]. Low mean intakes have also been reported by National Adults Nutrition Survey (NANS, 2008–2010), National Children’s Food Survey (NCFS, 2003–2004) and National Teens’ Food Survey (NTFS, 2005–2006) in Ireland: 27.8 g/day for adults (only 19% satisfies recommendations) [21] and 18.5–23.2 g/day for children/adolescents (just 17–39% met recommendations) [22]. Similar findings have been reported for Australia [23] and UK [24], while in other countries, WG intakes are even lower. For example, in France, as emerged from Comportements et Consommations Alimentaires en France survey (CCAF, 2009–2010), averages are 4.7 g/day for adults/older adults and 4.1 g/day for children/adolescents [25]. Finally, the Italian National Food Consumption Survey (INRAN-SCAI, 2005–2006) reported average values of 3.7 g/day for adults/older adults and 2.1 g/day for children/adolescents [26]; as recently emerged from the Italian Nutrition and Health Survey (INHES, 2010–2013), only 27.2% of adults and 21.9% of children/adolescents consume WG foods (mainly bread) at least once per week [27].

## 2. Whole Grains: An Overview

### 2.1. Definition

Each country or organization uses different WG definitions that are constantly updated [1,28,29]. The widely used International Definition was provided in 1999 by American Association for Cereal Chemists (AACC), which states: “whole grain shall consist of the intact, ground, cracked or flaked caryopsis, whose principal anatomical components—the starchy endosperm, germ, and bran—are present in the same relative proportions as they exist in the intact caryopsis” [30]. As unambiguous definition is essential for dietary recommendations, nutritional research, flour manufacturing process and labeling, in 2010 the European HEALTHGRAIN Consortium has developed, in line with AACC, a new definition: “whole grain shall consist of the intact, ground, cracked or flaked kernel after the removal of inedible parts, such as the hull and husk. The principal anatomical components—the starchy endosperm, germ and bran—are present in the same relative proportions as they exist in the intact kernel. Small losses of components—that is, less than 2% of the grain/10% of the bran—that occur through processing methods consistent with safety and quality are allowed”. Moreover, due to different composition of WG products, HEALTHGRAIN proposed that a product is labelled as WG food if “one for which the product is made with >30% whole-grain ingredients on a dry-weight basis and more whole-grain ingredients than refined-grain ingredients” [29].

### 2.2. Chemical Composition of Cereal Kernels

**Figure 1:**
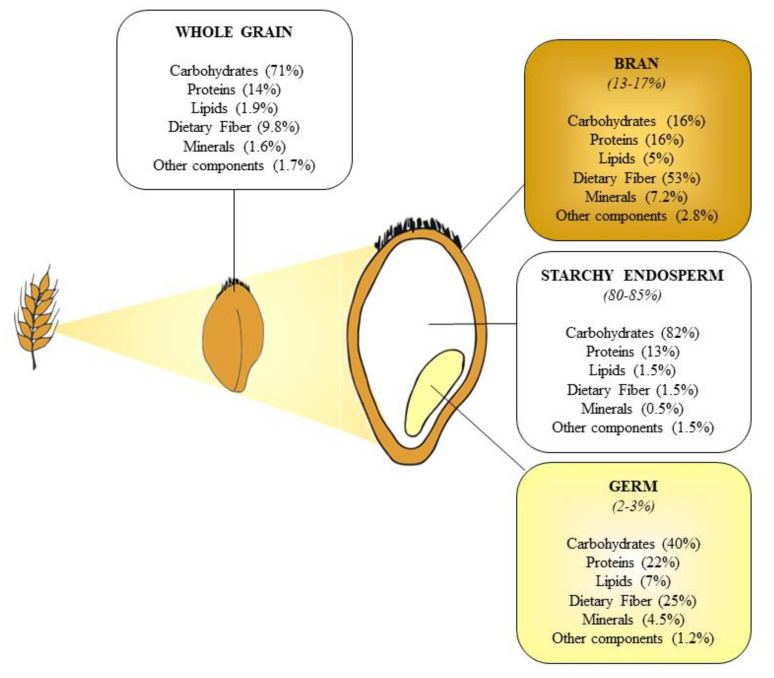
Nutritional composition of wheat kernel. Values are reported as percentage of dry matter.

Starchy endosperm, germ, and bran are the three principal fractions in cereal kernels [31]. Endosperm represents the most abundant fraction (constituting over 80% of caryopsis) containing large amounts of starch to supply energy and 75% of needed proteins for plant germination, some fiber, and micronutrients (especially iron, riboflavin, niacin, and thiamin). Germ (i.e., the embryo) occupies a small fraction of the seed (2–3% of kernel); particularly rich in proteins, fiber, and fats, it also contains significant amounts of mineral, B and E vitamins. Bran, consisting of multiple layers (pericarp, spermoderm, and perisperm), is the outer portion of the seed (13–17% of kernel); it contains fiber (more than 50%), proteins, starch (as “contamination” from endosperm), B vitamins, minerals, and several bioactive compounds, such as polyphenols (Figure 1) [31,32,33].

During milling process of refined flours, bran and germ are removed (and used for food and non-food applications) [33,34]; as a result, RG products contain fewer nutrients than WG counterparts. For example, in refined wheat flour, pantotenic acid, folate, iron and copper content and fiber are reduced, while some vitamins, potassium, magnesium, and manganese are even lost [35]; however, this loss can be compensated by fortifications, such as mandatory folate addition in USA [36].

### 2.3. Whole Grains and Health

As above mentioned, compared to refined counterparts, WG are associated with benefits for human health [30]. Among mechanisms of action, effects on postprandial glycemia, appetite and ad libitum energy intake have been proposed. In a randomized crossover study enrolling twenty young subjects (10 females and 10 males; Body Mass Index (BMI) = 21.7 ± 2.2 kg/m^2^), Kristensen and co-workers [37] reported that, with respect to refined counterparts, WG wheat bread ingestion led to increased satiety and reduced hunger, without modifying energy intake at the subsequent meals. The same group obtained similar results for WG pasta ingestion in overweight/obese (25 < BMI < 40 kg/m^2^) subjects [38]. Accordingly, WG food intake appears to be closely associated with reduced risk of obesity: cross-sectional dietary data from NHANES 2001-12 (which included 15,280 children and 29,683 adults) documented that WG intake inversely related to BMI, waist circumference and percentage of overweight/obese individuals [20]. Besides its beneficial role in obesity, WG consumption is closely associated with reduced risk of other chronic diseases, including cardiovascular disease, type II diabetes, metabolic syndrome, and several cancer types [8,10,11,12,13,14].

An important role in chronic diseases seems to be played by gut microbiota, whose composition is influenced not only by genetics and age, but also by diet [39]. A strong link between microbiota composition and food intake exists, as a consequence of long-term dietary habits [40]. In particular, high consumption of WG, vegetables and fruits is associated with greater microbial variety, while diet rich in RG and fats and low in fiber is associated with lower biodiversity [41,42]. An observational study has shown that high adherence to the Mediterranean diet (MD), a typical eating pattern of the Mediterranean basin characterized by high consumption of cereals, fruits, vegetables, and legumes, was associated with increased levels of anti-inflammatory compounds (such as short chain fatty acids, SCFAs) in fecal samples and reduced atherogenic compounds (such as trimethylamine *N*-oxide) in urine samples [43]. As MD recommends daily consumption of cereals, preferably as unrefined grains [44,45,46], it is conceivable that WGs cooperate with fruits and vegetables to change microbiota composition. Accordingly, in a randomized controlled, six-week trial, high WG consumption displayed better positive effects than high RG consumption, in terms of gut microbiota and immune responses [47].

In this context, it should be underlined that, unlike RG eaters, WG consumers generally follow health and diet recommendations and ingest few, if any, non-recommended, indulgent foods. For this reason, studies on WG intake must take into account all confounders, in order to remove potential bias from data.

## 3. Dietary Fiber and Polyphenols as Functional Compounds in Whole Grains

Fiber and polyphenols (or phenolic compounds) are the main dietary bioactive compounds studied for prevention of chronic diseases; they have different chemical structures, physical and biological properties, and ability to activate distinct metabolic pathways [48,49].

The definition of dietary fiber is constantly evolving and, although AACC has proposed that it is: “the remnants of the edible part of plants or analogous carbohydrates that are resistant to digestion and absorption in the human small intestine, with complete or partial fermentation in the large intestine” [50], the more recently accepted definition is that provided by Codex Alimentarius (CAC), i.e., “carbohydrate polymers with ten or more monomeric units, which are not hydrolyzed by the endogenous enzymes in the small intestine of humans” [51].

In WG, dietary fiber primarily derives from the outer portion of cereal kernel (although it can also be found in endosperm of some grains, like wheat and barley) and mainly consists of non-starch polysaccharides, distinguished by fermentability to SCFAs, solubility in water, viscosity, and cation exchange capacity [52,53]. Cellulose, galactomannans, xylans, xyloglucans, and lignin are part of insoluble dietary fiber, while arabinoxylans, arabinogalactans, β-glucans, and pectins of soluble dietary fiber [52]. Among cereals, wheat, triticale, and rye are rich in arabinoxylans, while oats and barley mainly contain β-glucans [54] (Table 1). As it will be discussed, high dietary fiber intake improves intestinal health, increases satiety, and reduces risk of some chronic diseases, including cancer [55,56].

Polyphenols, secondary metabolites found in plant tissues, are heterogeneous compounds, possessing one or more aromatic rings with one or more hydroxyl groups. Polyphenols can be subdivided into (i) phenolic acids, (ii) flavonoids, (iii) stilbenes, and (iv) lignans [59,64]; they can also be distinguished in soluble (free molecules in cell vacuoles) and insoluble (bound to cell wall elements, such as dietary fiber) compounds [32]. These phytochemicals are important for plant growth, defense, reproduction, and color; consequently, beyond genetics, also environmental factors significantly affect their levels, that vary greatly even between cultivars of the same species. Therefore, it is not possible to establish precise amounts of each compound in different plant-foods, and several polyphenols are still unidentified; therefore, literature data on polyphenol content in plant foods, including grains, is incomplete, difficult to compare and often contradictory. According to available data, WGs seem to contain polyphenol amounts similar to those found in fruits and vegetables, with some highly active phenolic compounds more represented (Table 1) [60,61,62,65,66]. Because of frequency of consumption [4], it has been estimated that WGs provide for about one-third of total polyphenol dietary intake [67].

Ferulic, *p*-cumaric, vanillic, siryngic, gallic, and caffeic acids are the most common phenolic acids of grains (Table 1) [60,61,62,65,66,68]. Some of them are present as esters or amides; this is the case of γ-oryzanol, a blend of ferulic acid esters and phytosterols, mostly found in rice [69] and avenanthramides, phenolic amides containing anthranilic acid and hydroxycinnamic acid moieties, exclusively found in oats [70]. Significant differences in phenolic acid amounts exist, depending on grain dimension and species, as well as on fiber type and content; ferulic acid, for example, is more abundant in smaller than in larger grains, and the higher the fiber content, the higher the ferulic acid content (Table 1) [61,71].

As above mentioned, WGs also represent a source of flavonoids, among which there are the two flavones apigenin and luteolin and the two flavanones naringenin and eriodictyol [60,63]. Additionally, anthocyanins have been reported in pigmented varieties of some WGs, such as barley, rice, rye, and wheat [63,72]; finally, among lignans, secoisolariciresinol is present in buckwheat and pinoresinol in oats [73].

Phenolic compounds might play a role in chronic diseases and, due to their antioxidant properties and ability to modulate specific signaling pathways involved in cell survival and death, are particularly beneficial in cancer [74,75,76]. However, physiological effects of these WG components strictly depend on their bioavailability, in turn influenced by binding to dietary fiber [77,78,79]. In cereals, most polyphenols (95%) are indeed covalently linked to polysaccharide chains of dietary fiber, mainly arabinoxylans [64]. As a consequence, although dietary fiber properties are generally attributed to non-starch polysaccharides, the “dietary fiber concept” is changing towards the “antioxidant dietary fiber concept” [80]. When gut microbiota ferments fiber, phenolic compounds are released into the intestinal lumen and absorbed by enterocytes. Non-fermented and non-absorbable polyphenols counteract the pro-oxidant effects of ingested foods, by scavenging free radicals [48], and meanwhile they synergize with bacteria-derived SCFAs in modulating cell death and differentiation [64,81]. Furthermore, dietary fiber-polyphenol association can downregulate energy metabolism, nuclear receptor signaling and lipid biosynthesis (via tumor necrosis factor-α and peroxisome proliferator-activated receptor-α), pathways involved not only in obesity, but also in cancer (especially of the gastrointestinal tract) [64,82,83].

## 4. Whole Grains and Gastrointestinal Cancers: An Overview

According to global cancer statistics, 19.3 million new cancer cases and 10 million all cancer deaths occurred in 2020 worldwide; more than one-third of cancer victims suffered from gastrointestinal tumors [84]. Based on molecular phenotype and histological characteristics, these tumors include cancers affecting upper and lower gastrointestinal tract, as well as salivary gland, liver and bile ducts, gallbladder, and exocrine pancreas [85]. Although a geographic description of cancer- and sex-specific incidence and mortality patterns exists, overall more than 60% of gastrointestinal cancer cases and deaths occurred in Asia, followed by Europe and North America [84].

Clinical management of gastrointestinal cancers remains a major challenge for clinicians, especially because most cases are diagnosed in advanced stages, when treatment options are limited [86]. A variety of etiological factors have been identified; it has been estimated that genetic defects account only for 5–10%, while harmful environmental conditions and unhealthy lifestyle represent 90–95% of risk factors [87]. Consequently, primary and secondary prevention strategies, including promotion of healthy lifestyle aimed at deeply modifying some risk behaviors (e.g., tobacco use, physical inactivity, unhealthy diet, and alcohol abuse), are particularly relevant for reducing cancer risk and outcomes.

Consumption of WGs is strongly recommended for gastrointestinal health. A large body of literature data concerning WG effects on gastrointestinal cancers are available, although WG action is not equal (and even absent) in different gastrointestinal organs. To the best of our knowledge, no epidemiological studies about WG intake and risk of gallbladder and bile duct carcinomas have been published, while only one study demonstrated inverse association between WG (and possibly bran and cereal fiber) intake and risk of hepatocellular carcinoma, the predominant histological form of primary liver cancer [88]. As emerged by a meta-analysis of case-control and cohort studies, high intake of WGs was also associated with reduced risk of pancreatic cancer [89]; nonetheless, lack of more prospective cohort studies prevents to draw robust conclusions.

Similarly, literature data on association between unrefined grains and oral cavity and oropharynx cancers are scarce, not updated and just based on few case-control and cohort studies. Some investigations highlighted that WG intake was favorably related to risk of upper aerodigestive tract cancers [90,91,92,93,94,95]. Conversely, other studies reported no [96,97,98,99] or even positive associations [100,101,102]. Due to these controversial results, data on WGs and oropharyngeal cancer risk are less consistent than those for other plant-derived foods. Finally, except for a large US prospective cohort study showing a marginal inverse relationship between WG food consumption and small intestinal cancer [103], also data referred to small bowel tumors are sparse and difficult to interpret.

Based on this evidence, we focused on colorectal, gastric, and esophageal tumors, the most diagnosed and severe gastrointestinal cancers, for which investigations are more extensive and continuously updated.

## 5. Whole Grains and Colorectal Cancer

In 2020, 1.9 million of individuals were diagnosed for colorectal cancer, the second mostly incident cancer and the third leading cause of cancer death worldwide. It has slightly higher incidence in males (1,065,960 cases) than females (865,360 cases) [84]; although incidence (10% of all cancer cases) is decreasing in developed countries, cases are increasing among younger adults, especially in USA [104].

Depending on location (proximal colon, distal colon and rectum), colorectal cancer varies in terms of etiology and sensitivity to specific risk factors [49,105]. Only 1–2% of cases have been associated with ulcerative colitis, Crohn disease and inflammatory conditions [106], while modifiable lifestyle factors, typical of industrialized countries (tobacco smoking, physical inactivity, red/processed meat and alcohol consumption, low intake of fruits and vegetables), are long-established risk factors [107].

In this context, WGs represent protective factors, as high intakes have been associated with significant decrease of cancer risk (Table 2). In a 14-year case-control study, conducted in Northern Italy and including 11,990 patients with several cancer types (among them, 955 colon and 625 rectum tumors), multivariate odd ratios (ORs) for the highest category of WG intake (>3 day per week) were 0.5 (95% CI 0.4–0.6) and 0.6 (95% CI 0.4–0.8) for colon and rectum cancers, respectively [108]. Intriguingly, Um and collaborators found sex-related differences in terms of WG association: the prospective CPS-II Nutrition Cohort study enrolling 50,118 men and 62,031 women (1742 incident colorectal cancer cases during the follow-up) found that the highest vs. lowest quintile of WG intake was associated with 23% and 43% lower risk of colorectal and rectal cancer, respectively, among men, but no association was found for women. Moreover, authors did not find any evidence of increased risk with consumption of RGs, grain-based sweets, or desserts [109]. Several meta-analyses have reinforced potential benefits of WGs against colorectal tumors [110,111,112,113]. For example, the World Cancer Research Fund International (WCRF) Continuous Update Project (CUP) has updated the systematic review and meta-analysis (until the end of May 2015) of prospective studies reporting 17% decreased risk for each 90 g/day WG increase [110]. Similarly, Schwingshackl and co-workers found 20% decreased risk of colorectal cancer with increasing WG intake up to 120 g/day [112]. Noteworthy, a recent meta-analysis from Zhang’s group found that WG/colorectal cancer association was significant only for sample size ≥500 [113]. Finally, as emerged from a Chinese 10-year follow-up study (enrolling 369 colon cancer subjects) high WG consumption (more than 17 g/day) also appears to be correlated with prognosis and survival rates [114]. Although all these findings highlight the positive role of WGs in cancer onset and/or outcomes, nonetheless no randomized clinical trials have tested the long-term impact of WG consumption on colorectal tumorigenesis up until now.

The American Institute for Cancer Research and the World Cancer Research Fund stated that eating at least 90 g/day WG reduces colorectal cancer risk, mainly due to its high fiber content [119]. Among mechanisms involved in WG protective effects, fiber-mediated reduction of fecal transit time, dilution, and removal of carcinogens (especially heterocyclic amines), maintenance of epithelial cell integrity and stimulation of bacterial fermentation (and, therefore, SCFA production that inhibits colon carcinogenesis) can be identified [120,121]. Accordingly, among all fiber containing foods, WGs are most consistently associated with incidence of colorectal cancer. Indeed, two large recent prospective US cohort studies did not find any association for total dietary fiber intake, but when different food sources were examined, lower risk for colorectal tumors was observed only in high cereal (especially unrefined) consumers. Moreover, such association was observed in men, but not in women; this sex-disparity might depend on lower fiber intake registered for women (mean fiber intake of 14 g/day for women and 20.0 g/day for men) [118]. Alegria-Lertxundi and co-workers [115] investigated the relationships between food groups, diet quality and colorectal cancer risk and reported no significant differences of intake between control and patient groups for the majority of food classes, except for lower WG intake (and higher egg consumption) in tumor cases; coherently, the observed protective effects of fiber-containing foods appeared to be mainly ascribed to WGs. A recent, large US cohort analysis (with more than 10,000 incident colorectal cases and more than 15 years of follow-up) further confirmed that fiber from grains, but not from other sources, was associated with lower incidence, especially for distal colon and rectal cancers [116]. High fiber and WG intake after diagnosis also leads to lower death rate, and this positive association again depends on fiber sources, with cereal fiber (especially from WG) showing the strongest link [117]. These data apparently disagree with the European Prospective Investigation into Cancer and Nutrition (EPIC) study that observed a significant lower risk of CRC in higher total fiber consumers [122,123]. Such a discrepancy may depend on less fiber in a typical American diet (with respect to the European one), as well as less proportional contribution of WG foods to total dietary fiber intake; indeed, about 39% of dietary fiber derives from grain foods containing no WGs, but RGs that have few amounts of fiber and are consumed in large quantities [124]. Therefore, further studies are necessary to evaluate dose-response relationship and influence of different fiber sources, taking into account that range of fiber intake widely varies depending on the examined population.

Concerning phenolic compounds, these phytochemicals exert anti-cancer activities in colon-cancer cells, mainly by inducing cell-cycle arrest and apoptosis. Just an example, ferulic and *p*-coumaric acids modulate S and G2/M phase transitions, respectively [125]; the two compounds also inhibit cancer cell proliferation, by inhibiting expression of epidermal growth factor receptor, one of the most relevant biomarkers in colorectal cancer [126], and related mitogenic signaling pathways [127,128]. Likewise, in human colon cancer cells, secoisolariciresinol diglycoside and its metabolites (enterolactone and enterodiol) induce S-phase cell cycle arrest, by modulating key regulatory proteins (cyclin A and cyclin-dependent kinase 4) [129,130,131]. By possessing estrogenic activity, some flavonoids (such as apigenin, naringenin, luteolin, and eriodictyol) contribute to colon cancer prevention, through activation of estrogen receptor-β in colonocytes [132,133,134,135]. Finally, some miRNAs involved in colorectal cancer are sensitive to phenolic compounds: for example, miRNA384 is up-regulated by luteolin, thus resulting in decreased expression levels of pleiotrophin, a cytokine upregulated in colorectal tumors [136,137,138].

In order to overcome challenges in polyphenol delivery to target tissues, recent studies have attempted to find novel strategies for improving bioavailability and anti-tumor efficacy of these phytochemicals. For example, the novel stable ferulic derivative tributyltin(IV) ferulate has been designed and found to potently exert anti-tumor activity; this synthetic compound, indeed, triggers autophagic cell death through generation of reactive oxygen species and endoplasmic reticulum stress in colon cancer cells [139]. Similarly, a novel nanoparticle system, consisting of encapsulated gallic acid and gum arabic as coating material, has shown promising anti-cancer properties: the formulated nanoparticles, indeed, were selectively internalized by cancer cells, thus exerting potent anti-oxidant and anti-neoplastic effects, as assessed by cytotoxic, migration, and apoptosis assays [140].

## 6. Whole Grains and Gastric Cancer

Gastric cancer is the fourth cause of tumor-related deaths. Incidence (5.6% of all cancer cases) is higher in males (719,523 cases) than females (369,580 cases); 75.3% of cases occur in Asia, followed by Europe (12.5%) and Latin America and Caribbean (6.2%) [84].

Generally, gastric cancer is classified into non-cardiac gastric cancer, originating from distal regions of stomach, and cardiac gastric cancer, arising near the esophageal-gastric junction [141]; both forms are associated with cigarette smoking and *Helicobacter pylori* infection, while cardiac gastric cancer is also related to other risk factors, including esophageal reflux, Barrett’s esophagus, and obesity [142,143,144,145,146,147,148,149].

Among factors affecting cancer onset, dietary habits play an important role [150,151,152]: salt-preserved foods and smoked meats potentiate carcinogenic effects of *H. pylori* infection [153], whereas fruits, vegetables, and WGs are protective factors [154,155]. A prospective population-based case-control study reported in men, but not in women, a modestly lower risk of stomach cancer with diet patterns high in WGs, only when combined with citrus fruit and vegetables [156]. Several meta-analyses have been published on WG/gastric cancer association, relying primarily or entirely on case-control studies and without dose-response analyses. However, all studies reported that increasing WG consumption was notable in showing a negative association with stomach cancer risk (ranging from 13 to 50% lower risk for highest WG consumers) and/or RG intake generally appeared to be a dose-dependent risk factor (63–65% increment of the risk) (Table 3) [92,157,158,159,160,161,162,163]. However, it should be recalled that RG-rich diet is usually poor in WGs (and other dietary fiber sources) and associated with unfavorable lifestyles. Therefore, for gastric cancer, nutritional and lifestyle combination, rather than RG alone, may account for direct associations observed in the studies.

To date, no conclusions on the role of fiber in WG/gastric cancer association can be drawn since available investigations are somehow misleading and difficult to interpret. Except for the cohort Iowa Women’s Health Study (demonstrating strong protective effects of WG fiber against stomach cancer) [95], almost all studies consider only total dietary fiber intake and/or report no association at all for fiber from grains [55,161,162].

Among polyphenols, gallic acid has been shown to inhibit *H. pylori* proliferation, as well as invasion and metastasis of cancer cells [164]. Similarly, Ho and colleagues [165] demonstrated that gallic acid can in vitro reduce migration of human gastric carcinoma cells, through inhibition of RhoB expression and modulation of Akt signaling. Polyphenols also activate apoptosis: caffeic acid induces cell death by modulating cellular Ca^2+^ homeostasis [166], ferulic acid activates caspase-3 and caspase-9 [75], and apigenin modulates expression of pro- (Bax and caspase-3) and anti-apoptotic (Akt and Bad) proteins [167,168]. Lastly, polyphenols are able to modulate activity of specific miRNAs: luteolin inhibits Bcl-2 expression by upregulating miR-34a, while *p*-coumaric acid exerts antitumor effects by regulating hsa-miR-30a-5p, hsa-miR-125a-5p, and hsa-miR-7-5p [169,170,171,172].

## 7. Whole Grains in Esophageal Cancer

According to Globocan 2020, 508,585 cancer victims (5.3% of all cancer cases) were affected by esophageal cancer, the sixth cause of cancer deaths [84]. Incidence of esophageal cancer (3.1% of all cases) is higher in males than females; the highest mortality (78.2%) is registered in Asian continent [84].

Esophageal tumors are distinguished into esophageal squamous cell carcinomas, affecting upper layer cells lining esophagus, and adenocarcinomas, arising in glandular cells located between the esophagus–stomach junction [173,174]; esophageal squamous cell carcinomas are more frequent in developing countries, while esophageal adenocarcinomas predominate in eastern Asia and Africa [175]. Distinct risk factor profiles have been identified: tobacco smoking and alcohol abuse are main risk factors for esophageal squamous cell carcinoma, while obesity and gastro-esophageal reflux disease are key risk factors for adenocarcinoma. Specific dietary items and nutrients impact risk of both types of cancer [176]: for example, red, pork and processed meat, moldy food and pickled vegetable consumption are risks attributable to the entire population, while more varied diet, raw and cooked vegetables, vitamins, fiber, and carbohydrates are included among protective dietary factors [157,177,178,179,180].

Higher frequency of WG food consumption may be accounted among indicators of reduced risk of esophageal cancer (Table 4). In a small case-control study, for example, Levi and co-workers reported significant decrease in cancer risk in individuals consuming high amounts of WG foods (whole wheat bread and cereals), while cancer onset was directly related to consumption of RG items (white bread and biscuits, pizza, pasta, and rice) [91]. Decreased risk for high WG intake has been reported by retrospective and prospective studies, although with different ratios: for example, the above mentioned Italian case-control study from La Vecchia’s group [108] reported 60% decreased risk for the highest WG intake, while the recent HELGA cohort study from Skeie and co-workers showed 35–45% reduction [181]. Noticeably, authors observed that such association varied with cereals and food products, with WG wheat and bread being associated with lower risk. This finding can be explained considering that cereals and cereal-based foods have different composition and concentration of nutrients and bioactive compounds, which cooperate to exert positive effects [182,183]. In this context, dietary fiber may play a crucial role, as inverse correlation exists between dietary fiber intake and risk of both Barrett’s esophagus, an intermediate pre-neoplastic lesion, and esophageal cancer [180,184]. Potential mechanisms of protective action include modification of gastroesophageal reflux and/or weight control, neutralization of carcinogens contained in food, amelioration of cancer-associated esophageal dysbiosis, and direct action on cancer cells [180,184,185,186,187,188]. The prospective 14-year Iowa Women’s Health Study, enrolling a cohort of 34,651 post-menopausal, initially cancer-free women, reported that malignancy incidence was inversely associated with WG intake, as well as with total fiber intake. In this context, some interesting data emerged: (i) none of inverse associations observed for fruit fiber, vegetable fiber, and total grain fiber was statistically significant; (ii) no protective effect was found for fiber from RGs (according to the evidence that milling process lowers content of fiber and bioactive compounds); (iii) the relationship with dietary fiber was driven by strong inverse association for WG fiber [95]. In the light of these findings, it should be advised to distinguish WGs or RGs as source of fiber, in order to avoid biased data [55,180].

Additionally, polyphenols could be beneficial in esophageal cancer, thanks to their antioxidant activity, ability to improve esophageal reflux-related inflammation, and modulation of cell proliferation and survival [189,190]. Gallic acid, for example, induces cell death in human squamous esophagus carcinoma cells, much likely by activating both extrinsic and intrinsic apoptotic pathways, as well as by downregulating the Akt/mTOR survival signaling cascade [191]. Recently, protective roles of apigenin have been confirmed in esophageal tumors: in in vitro and in vivo experimental models, this flavonoid has been reported to (i) induce apoptosis of tumor cells, (ii) inhibit tumor-dependent angiogenesis, and (iii) attenuate inflammatory responses, by inhibiting gene expression of the pro-inflammatory cytokine interleukin-6, whose levels are elevated in tumor tissues [192,193].

## 8. Conclusions and Future Perspectives

Cancer onset, progression, and outcome are strictly dependent on interaction among genetic, metabolic, and environmental factors. Remarkably, besides some unhealthy habits (such as tobacco use, alcohol abuse, and sedentary lifestyle), consumption of harmful foodstuff and nutrients increases cancer risk; coherently, healthy dietary behaviors, which involve consumption of healthy foods (fruits, vegetables, cereals, legumes, fish, olive oil) and nutrients (antioxidants, phytochemicals, fiber, vitamins, mono- and poly-unsaturated fatty acids), are worldwide recognized as a valid strategy for primary cancer prevention. Scientific organizations of several countries encourage WG intake in maintaining health and reducing risk of chronic diseases, such as type 2 diabetes, cardiovascular disease, and cancer [119,194,195,196,197]. It has been estimated, in fact, that low WG intake resulted in almost 270,000 avoidable deaths and almost 4 million disability-adjusted life years in the European Union in 2015 [198].

Herein, we focused on inverse relationship between WGs, whose consumption is increasingly recommended, and gastrointestinal cancer onset and outcomes. What emerged is that WGs, unlike refined counterparts, consistently protect against gastrointestinal cancer, especially colorectal type; such differences can mainly be ascribed to reduction (or loss) of beneficial nutrients and phytochemicals during milling process. WG is indeed a complex food matrix containing different bioactive compounds, which synergistically act in chronic disease prevention. It is therefore difficult to identify which constituent is responsible for protection; for this reason, attention should be shifted not to single compounds, but instead to WG food matrix. For example, some WG positive effects essentially depends on fiber, but fiber varies from grain to grain and is present in other food items (vegetable, fruits, and legumes) that are equally consumed by high WG users. Thereby, although both fiber and WGs have healthy benefits, they are not interchangeable and consumers should pay particular attention to high-fiber products, sometimes containing bran or other added fiber without actually having much, if any, WG.

Noteworthily, WG consumers are more likely to consume less sugar, alcohol, fat, red meat, and indulgent foods, while consuming more fruits, vegetables, and fish; moreover, they have high education and socioeconomic status, as well as healthy lifestyle (physically active, no smoking). For this reason, most of studies investigated WG effects after accurate statistical adjustments for all of these possible confounders, thus removing potential bias from data and providing authentic and real relationship between WG intake and gastrointestinal cancer. Nonetheless, several elements point out that we are far from a solid, scientific-based knowledge for developing individualized WG-based regimens to prevent and manage cancer. WG consumption, indeed, reduces risk of digestive tract tumors with significant heterogeneity because of additional confounding factors, including differences in (i) type, duration, quality, and sample size of investigations; (ii) methods of collecting WG intake (food-frequency questionnaires vs. more quantitative questionnaires); (iii) type of WG foods; (iv) racial and ethnic demographic groups displaying different nutritional habits.

Despite all these limitations, beneficial effects of WGs cannot be denied, and therefore programs aimed at increasing WG consumption should be implemented through a broad partnership involving both public (Government authorities) and private (industries) stakeholders. Several barriers to WG consumption should, indeed, be removed by effective strategies: (i) univocal, quantitative, and international recommendations; (ii) nutritional education programs; (iii) improvement of sensory characteristics and increase of variety of WG foods (in order to satisfy different eating habits of consumers of all ages); (iv) better identification of WG-containing products; (v) reduction of purchase costs.

## Figures and Tables

**Table 1:** Content of polyphenols and fiber in some whole grains commonly consumed worldwide.

Compound ^1^	Wheat	Oat	Corn	Rice	Refs
Dietary fiber	9.7–13.1	7.6–10.6	2–7.3	1.4–3.75	[57,58]
Total polyphenols	538	471.7	497.1	421.8	[59]
Total phenolic acids	1342 (75%) ^2^	472 (75%) ^2^	601 (85%) ^2^	197–376 (62%) ^2^	[60,61]
Ferulic acid	11.6–870	249.4–1044.9	97–584.0	68.2–301.7	[62]
*p*-coumaric acid	3.5–293.0	607.3	97.0–584.0	22.8–85.0	[62]
Gallic acid	6.5–195.0	1.7–241.2	0.5–116.5	5.5–115.6	[62]
Caffeic acid	0.5–51.9	3.6–9.2	5.7–24.4	1.0–3.5	[62]
Total flavonoids	95.8–212	n.r.	607.1–1277	94–3274	[63]

^1^ All data are expressed as μg/g dry weight, except for dietary fiber, expressed as g/100 g of grain and total polyphenols, expressed as mg of Gallic Acid Equivalent/100 g dry weight. ^2^ Percentage of bound form. n.r.: not reported by authors.

**Table 2:** Some epidemiological studies on whole grains/whole grain fiber and colorectal cancer.

	Study Type and Design	Main Findings *	Refs
**Whole grains**	14-year US prospective population-based case-control study (112,149 participants (1742 CRC) from the Cancer Prevention Study-II Nutrition Cohort 1999–2013) Quintiles of WG intake (g/day): Q1: <19 for men; <18 for women Q5: 116.7–1296 for men; 117.1–1255 for women	Similar WG intake in women (mean: 72.8 g/day; 10th–90th percentile distribution: 10.6–168 g/day) and men (mean: 74.5 g/day; 10th–90th percentile distribution: 9.2–174 g/day) High WG intake associated with low CRC risk among older men, but not women (HR = 0.77, 95% CI 0.61–0.97; *p* = 0.03 for men; HR = 1.10, 95% CI 0.88–1.36; *p* = 0.14 for women; *p* interaction by sex = 0.01) Men in the highest quintile: 43% reduced risk (HR = 0.57, 95% CI 0.35–0.93, *p* = 0.04) No association of RG with CRC risk	[109]
	10-year prospective population study (369 CRC patients, 154 deaths during the follow-up) Quartiles of WG intake (g/day) Q1: ≤7.1 Q2: 7.1–10.7 Q3: 10.7–17.9 Q4: >17.9	High WG intake associated with risk of mortality (HR_Q4_ vs. _Q1_ = 0.56, 95% CI 0.35–0.89; *p* for trend 0.05)	[114]
	Meta-analysis of 11 prospective studies for WG consumption and 3 reports for RG consumption	Inverse association between CRC risk and WG intake (RR = 0.88, 95% CI 0.83–0.94, *I*^2^ = 35%, *p* = 0.13) (10 studies with 9223 CRC cases; overall intake range: 0–374 g/day) Each additional daily 30 g of WGs inversely associated with CRC risk (RR = 0.95, 95% CI 0.93–0.97, *I*^2^ = 58%, *p* = 0.02); 20% decreased risk with WG intake up to 120 g/day No association for RG intake (RR = 1.46, 95% CI 0.80–2.67, *I*^2^ = 71%, *p* = 0.06) (900 CRC cases, overall intake range: 15–585 g/day)	[112]
	Meta-analysis of 34 studies of WG intake and risk of digestive tract cancer [CRC: 7 case-control and 10 cohort studies (1,489,581 participants and 19,424 cases)]	Inverse association between CRC risk and WG intake (RR = 0.89, 95% CI 0.84–0.93; *p* < 0.001; *I*^2^ = 38.2%, *p* = 0.029). Positive effects of WGs only in studies with sample size ≥500 (RR = 0.91, 95% CI 0.88–0.94, *p* < 0.001) No statistically significant heterogeneity in women (*I*^2^ = 0%, *p* = 0.619), European (*I*^2^ = 0%, *p* = 0.732), before 2010 publication year (*I*^2^ = 0%, *p* = 0.622) and adjustment for energy (*I*^2^ = 4.6%, *p* = 0.399) studies	[113]
**Whole grains/whole grain fiber**	Spanish observational case-control study (308 CRC and 308 controls) Tertiles of WG fiber intake not defined, but referred to Healthy Eating Index for Spanish Diet (HEISD) (T1: 69; T2: 69–74.5; T3: >74.5) and MedDietScore (MDS) (T1: <35; T2: 35–37; T3: >37)	WG intake lower in CRC patients than controls (14.4 ± 19.9 vs. 18.8 ± 23.4 g/day, *p* = 0.012). Inverse association between WG intake and CRC risk (OR_T3 vs. T1_ = 0.62, 95% CI 0.39–0.98) Consumption of fiber-containing foods, especially WG, associated with lower CRC risk (OR_T3 vs. T1_ = 0.65, 95% CI 0.35–1.21).	[115]
	US Prospective NIH-AARP Diet and Health Study (1995–2011) including 478,994 subjects (285,456 men and 193,538 women) cancer free at the beginning; 10,200 incident cases (6712 men and 3488 women) at the end. Quintiles of WG intake (servings/1000 kcal/day) Q1: 0.2 Q2: 0.4 Q3: 0.6 Q4: 0.8 Q5: 1.8 Quintiles of WG fiber intake (g/1000 kcal/day) Q1: 1.7 Q2: 2.5 Q3: 3.2 Q4: 4.0 Q5: 5.7	Positive association for both WGs (HR_Q5 vs. Q1_ = 0.69, 95% CI 0.64–0.73; *p* < 0.001) and dietary fiber (HR_Q5 vs. Q1_ = 0.70, 95% CI 0.66–0.75; *p* < 0.0001) After adjustment for potential confounders: HR_Q5 vs. Q1_ = 0.83 (95% CI 0.78–0.89; *p* < 0.001) for WGs and HR_Q5 vs. Q1_ = 0.92 (95% CI 0.86–0.99; *p* < 0.03) for dietary fiber intake. The association remained statistically significant after adjustment for folate (HR_Q5 vs. Q1_ = 0.84, 95% CI 0.79–0.90; *p* < 0.001) and dietary fiber intake (HR_Q5_ vs. _Q1_ = 0.84, 95% CI 0.78–0.90; *p* < 0.001) Only fiber from grains was inversely associated with CRC (HR_Q5 vs. Q1_ = 0.89, 95% CI 0.83–0.96; *p* < 0.001) No sex-dependence (*p* = 0.13 for interaction)	[116]
	963 US females from Nurses’ Health Study cohort (NHS; 1980–2010) and 612 US males from Health Professionals Follow-up Study cohort (HPFS; 1986–2010) diagnosed stage I to III CRC throughout follow-up. Quintiles of WG fiber intake (g/1000 kcal/day) Q1: 1.7 Q2: 2.5 Q3: 3.2 Q4: 4.0 Q5: 5.7	WG intake associated with low CRC-specific mortality (HR per 20 g/day increment = 0.72, 95% CI 0.59–0.88; *p* = 0.002), also after adjusting for fiber intake (HR = 0.77, 95% CI 0.62–0.96; *p* = 0.02), and all-cause mortality (HR = 0.88, 95% CI 0.80–0.97; *p* = 0.008 for trend). Cereal fiber intake associated with low CRC-specific mortality (HR per 5 g/day increment = 0.67, 95% CI 0.50–0.90; *p* = 0.007) and all-cause mortality (HR = 0.78, 95% CI 0.68–0.90; *p* < 0.001). Vegetable fiber associated with low all-cause mortality (HR = 0.83, 95% CI 0.72–0.96; *p* = 0.009), but not CRC-specific mortality (HR = 0.82, 95% CI 0.60–1.13; *p* = 0.22); no association for fruit fiber. Patients with increased fiber intake after diagnosis: lower mortality rate [each 5 g/day increase associated with 18% decrease in CRC-specific mortality (95% CI 7–28%; *p* = 0.002) and 14% decrease in all-cause mortality (95% CI 8–19%; *p* = 0.001)].	[117]
	1902 US females from Nurses’ Health Study cohort (NHS; 1980–2012) and 1276 US males from Health Professionals Follow-up Study cohort (HPFS; 1986–2012) diagnosed CRC throughout follow-up. Deciles of total fiber intake (g/day): D1: 9.56 in women; 13.1 in men D10: 24.8 in women; 33.7 in men Deciles of cereal/WG fiber intake (g/day): D1: 1.60/6.54 in women; 2.58/9.70 in men D10: 7.43/39.01 in women; 12.0/58.3 in men Deciles of fruit/vegetable fiber intake (g/day): D1: 1.03/2.79 in women; 1.10/3.30 in men D10: 8.50/9.87 in women; 10.2/13.1 in men	No association between total fiber and CRC risk. No association for fruit or vegetable fiber. Inverse association between cereal fiber intake and CRC risk only in men (HR_D10 vs. D1_ = 0.75, 95% CI 0.57–1.00). Inverse association between intake of WG fiber and risk of CRC only in men (HR_D10 vs. D1_ = 0.72, 95% CI 0.54–0.96).	[118]

* Findings on WG intake per se, after adjusting for confounding factors (e.g., age, sex, education, smoking, dietary habits, alcohol, physical activity, etc.) through multivariate models. CI: Confidence interval; CRC: colorectal cancer; HR: Hazard Rate; OR: Odd ratio; RG: refined grain; RR: Relative Risk; WG: whole grain.

**Table 3:** Some epidemiological studies on whole grains/whole grain fiber and gastric cancer.

	Study Type and Design	Main Findings *	Refs
**Whole grains**	Prospective 14-year population-based case-control Cancer Prevention Study [533,391 women (439 deaths for GC) and 436,654 men (910 deaths for GC)] Tertiles of WG intake (days/week): T1: < 1 T2: 1–4 (4.5 for women) T3: > 4 (4.5 for women)	Men: high WG consumption associated with decreased risk only in age-adjusted model (RR_T2 vs. T1_ = 0.87, 95% CI 0.74–1.03; RR_T3 vs. T1_ = 0.77, 95% CI 0.66–0.90; *p* < 0.001), but not in multivariate-adjusted model (RR_T2 vs. T1_ = 0.94, 95% CI 0.79–1.11; RR_T3 vs. T1_ = 0.90, 95% CI 0.77–1.06; *p* = 0.17). More than 4 times/week cold cereal intake related to lower risk with respect to low (<once/week) intake (RR = 0.83, 95% CI 0.68–1.00; *p* = 0.03 for trend). Men with positive family GC history, consuming WG products >4 days/week, showed lower risk (RR = 0.31, 95% CI 0.15–0.64) with respect to men with no family GC history. Women: no association between WGs and GC risk. Women consuming brown rice, whole wheat or barley 6 to 7 times/week were at greater risk of fatal stomach cancer with respect to women with no intake (RR _T3 vs. T1_ = 1.41, 95% CI 1.04–1.91; *p* for trend = 0.05).	[156]
	Retrospective 10-year hospital-based case-control study (745 GC patients and 3526 controls) Tertiles of WG food intake (simple score of consumption): T1: low T2: intermediate T3: high Tertiles of RG food intake (portions/week): T1: 0–14 T2: 15–21 T3: ≥22	Whole meal consumption negatively correlated with GC risk For WG foods: OR_T3 vs. T1_ = 0.5, 95% CI 0.4–0.7 For RG foods: OR_T2 vs. T1_ = 1.24, 95% CI 1.0–1.5 and OR_T3 vs. T1_ = 1.54, 95% CI 1.2–2.0	[92]
	Retrospective 3-year hospital-based case-control study (143 GC patients and 328 controls) Tertiles of whole-meal bread intake (simple score of consumption): T1: low T2: intermediate T3: high	Whole meal consumption negatively correlated with GC risk RR_T2 vs. T1_ = 1.26, 95% CI 0.79–2.01 RR_T3 vs. T1_ = 0.48, 95% CI 0.28–0.82	[160]
	Meta-analysis of 5 hospital-based case-control, 4 population-based case-control and 2 prospective cohort studies (2920 GC cases and 527,256 controls)	WG consumption inversely related to GC in Europe (OR = 0.72, 95% CI 0.19–1.24) and America (OR = 0.61, 95% CI 0.38–0.85), both in hospital-based case-control (OR = 0.50, 95% CI 0.35–0.65) and cohort (OR = 0.61, 95% CI 0.38–0.85) studies	[158]
	Meta-analysis of 34 studies of WG intake and risk of digestive tract cancer [GC: 9 case-control and 2 cohort studies (1,021,955 participants and 8274 GC cases)]	WG consumption: 36% decrease in GC risk (RR = 0.64, 95% CI 0.53–0.79; *p* < 0.001), with a significant heterogeneity (*I*^2^ = 78.2%, *p* = 0.001) WG intake was a protective factor for case-control (RR = 0.55, 95% CI 0.41–0.74; *p* < 0.001) and European (RR = 0.64, 95% CI 0.53–0.79; *p* < 0.001) studies No significant association in cohort (RR = 0.89, 95% CI 0.78–1.01; *p* = 0.070) and American (RR = 0.70, 95% CI 0.50–1.00; *p* = 0.051) studies	[113]
	Meta-analysis of 19 studies (17 case-control and 2 cohort studies; 994,258 participants) Consumption of WGs or RGs: Low: <1/month Moderate: 1–2 times/week High: >3 times/week	WG consumption: 13% decrease in GC risk (OR = 0.87, 95% CI 0.79–0.95; *p* = 0.003) High consumption: 44% reduced risk (OR _high vs. low_ = 0.56, 95% CI 0.45–0.69; *p* < 0.001) No significant correlation for moderate consumption RG consumption: 36% increase in GC risk (OR = 1.36, 95% CI 1.21–1.54; *p* < 0.001) 63% increased GC risk in high consumers (OR = 1.63, 95% CI 1.49–1.79; *p* < 0.001) 28% increased GC risk in moderate consumers (OR = 1.28, 95% CI 1.18–1.39; *p* < 0.001) 53% increased GC risk in rice consumers (OR = 1.53, 95% CI 1.31–1.79; *p* < 0.001) 28% increased GC risk in RG, not-rice consumers (OR = 1.28, 95% CI 1.11–1.49; *p* = 001) No correlation between small amounts of RG intake and GC risk	[159]
**Whole grain fiber**	Prospective 14-year cohort Iowa Women’s Health Study (34,651 initially free-cancer women; 56 GC) Tertiles of WG fiber intake (g/day): T1: 0–1.49 T2: 1.50–3.98 T3: 3.99–35.75 Tertiles of RG fiber intake (g/day): T1: 0–1.37 T2: 1.37–2.35 T3: 2.35–16.93	WG fiber intake inversely related to GC risk (HRR_T3 vs. T1_ = 0.53) No association for RG fiber intake	[95]

* Findings on WG intake per se, after adjusting for confounding factors (e.g., age, sex, education, smoking, dietary habits, alcohol, physical activity, etc.) through multivariate models. CI: Confidence Interval; GC: gastric cancer; HRR: Hazard Rate Ratio; OR: Odds Ratio; RG: refined grain; RR: Relative Risk; WG: whole grain.

**Table 4:** Some epidemiological studies on whole grains/whole grain fiber on esophageal cancer.

	Study Type and Design	Main Findings *	Refs
**Whole grains**	Swiss 7-year retrospective hospital-based case-control study (349 controls and 101 EC patients). Whole (whole wheat bread and cereals) and refined (white bread and biscuits, pizza, pasta and rice) grain foods Tertiles for WG intake (times/week): T1: <4 T2: 4–10 T3: >10 Tertiles for RG intake (times/week): T1: <9 T2: 9–17 T3: 17	EC risk inversely correlated to WG intakes (OR_T3 vs. T1_ = 0.30, CI 95% 0.1–0.6) and directly correlated to RG intakes (OR_T2 vs. T1_ = 2.6, CI 95% 1.1–6.2; OR_T3 vs. T1_ = 3.7, CI 95% 1.8–7.9)	[91]
	Italian 14-year hospital-based case-control studies (1983–1997) 10058 controls and 11.990 cancer patients (410 EC cases). Tertiles for WG food intake (day/week): T1: no or rare consumption T2: 1–3 T3: >3	WG consumption associated with reduced risk (OR _T3 vs. T1_ = 0.4, 95% CI 0.2–0.7 and OR_T2 vs. T1_ = 0.4, 95% CI 0.3–0.7)	[108]
	Meta-analysis of 34 studies of WG intake and risk of digestive tract cancer (EC: 4 case-control studies and 2 cohort studies (151,742 participants and 1223 EC cases))	WG consumption associated with reduced risk (RR = 0.54, 95% CI 0.44–0.67, *p* < 0.001) No statistically significant heterogeneity (*I*^2^ = 27.7%, *p* = 0.217)	[113]
	Scandinavian 11-year prospective population-based case-control study (113,993 members from HELGA cohort including 56 EAC patients and 54 ESCC patients; 73.2% male and 33.8% women) Sex-specific tertiles of total WG intake (g/day): T1: F: 0–37.6; M: 0–37.8 T2: F: 37.7–60.5; M: 37.9–62.1 T3: F: 60.6–160.0; M: 62.2–160.0 Sex-specific tertiles of WG wheat intake (g/day): T1 F: 0–6.1, M: 0–2.5 71 T2 F: 6.2–32.1, M: 2.6–8.0 T3 F: 32.2–94.0, M: 8.1–65.7 Sex-specific tertiles of WG bread intake (g/day): T1: 0–59.6 for men; 0–65.8 for women T2: 62.0–125.0 for men; 68.2–113.2 for women T3: 129.6–520.0 for men; 113.8–520.0 for women	Inverse correlation between EC risk and total WGs (HR _T3 vs. T1_ = 0.55, 95% CI 0.31–0.97) and WG products (HR _T3 vs. T1_ = 0.51, 95% CI 0.30–0.88 per 25 g) Only wheat showed significant associations in adjusted models (adjusted HR_T3 vs. T1_ = 0.32, 95% CI 0.16–0.63) Only WG bread showed significant associations in adjusted model (adjusted HR _T3 vs. T1_ = 0.88, 95% CI 0.80–0.96 per 25 g WG bread) EAC: adjusted HR = 0.81, 95% CI 0.65–1.02 per 50 g WG products and HR = 0.85, 95% CI 0.66–1.09 per 20 g WGs ECCS: adjusted HR = 0.66, 95% CI 0.51–0.86 per 50 g WG products and adjusted HR = 0.75, 95% CI 0.56–1.00 per 20 g WGs	[181]
**Whole grains/whole grain fiber**	Prospective 14-year cohort Iowa Women’s Health Study (34,651 initially free-cancer women; 21 EC and 56 GC) Tertiles of WG intake (servings/week): T1: 0–6.5 T2: 6.9–12.5 T3: 13.0–108.5 Tertiles of WG fiber intake (g/day): T1: 0–1.49 T2: 1.50–3.98 T3: 3.99–35.75	Inverse correlation between EC risk and WG (HRR _T3 vs. T1_ = 0.47) or WG fiber (HRR _T3 vs. T1_ = 0.35) intake	[95]

* Findings on WG intake per se, after adjusting for confounding factors (e.g., age, sex, education, smoking, dietary habits, alcohol, physical activity, etc.) through multivariate models. CI: Confidence Interval; EAC: esophageal adenocarcinoma; EC: esophageal cancer; ESCC: esophageal squamous cell carcinoma; HR: Hazard Rate; HRR: Hazard Rate Ratio; OR: Odds Ratio; RG: refined grain; RR: Relative Risk; WG: whole grain.
